# A population-scale temporal case–control evaluation of COVID-19 disease phenotype and related outcome rates in patients with cancer in England (UKCCP)

**DOI:** 10.1038/s41598-023-36990-9

**Published:** 2023-07-25

**Authors:** Thomas Starkey, Maria C. Ionescu, Michael Tilby, Martin Little, Emma Burke, Matthew W. Fittall, Sam Khan, Justin K. H. Liu, James R. Platt, Rosie Mew, Arvind R. Tripathy, Isabella Watts, Sophie Therese Williams, Nathan Appanna, Youssra Al-Hajji, Matthew Barnard, Liza Benny, Alexander Burnett, Jola Bytyci, Emma L. Cattell, Vinton Cheng, James J. Clark, Leonie Eastlake, Kate Gerrand, Qamar Ghafoor, Simon Grumett, Catherine Harper-Wynne, Rachel Kahn, Alvin J. X. Lee, Oliver Lomas, Anna Lydon, Hayley Mckenzie, NCRI Consumer Forum, Emma Kinloch, Emily Lam, Gillian Murphy, Malcolm Rhodes, Kate Robinson, Hari Panneerselvam, Jennifer S. Pascoe, Grisma Patel, Vijay Patel, Vanessa A. Potter, Amelia Randle, Anne S. Rigg, Tim M. Robinson, Rebecca Roylance, Tom W. Roques, Stefan Rozmanowski, René L. Roux, Ketan Shah, Remarez Sheehan, Martin Sintler, Sanskriti Swarup, Harriet Taylor, Tania Tillett, Mark Tuthill, Sarah Williams, Yuxin Ying, Andrew Beggs, Tim Iveson, Siow Ming Lee, Gary Middleton, Mark Middleton, Andrew Protheroe, Tom Fowler, Peter Johnson, Lennard Y. W. Lee

**Affiliations:** 1 Institute of Cancer and Genomic Sciences, University of Birmingham, Birmingham, UK; 2 UK Health Security Agency, London, UK; 3 University Hospitals Birmingham NHS Foundation Trust, Birmingham, UK; 4 Oxford University Hospitals NHS Trust, Oxford, UK; 5 Cancer Institute, University College London, London, UK; 6 University of Leicester, Leicester, UK; 7 University of Leeds, Leeds, UK; 8 Leeds Institute of Medical Research at St James’s, Leeds, UK; 9 Torbay and South Devon NHS Foundation Trust, Torquay, UK; 10 Queen Elizabeth Hospital Birmingham, Birmingham, UK; 11 Royal Free London NHS Foundation Trust, London, UK; 12 University of Sheffield, Sheffield, UK; 13 Oxford Medical School, University of Oxford, Oxford, UK; 14 Birmingham Medical School, University of Birmingham, Birmingham, UK; 15 Weston Park Cancer Centre, Sheffield, UK; 16 Department of Oncology, University of Oxford, Oxford, UK; 17 Taunton and Somerset NHS Foundation Trust, Taunton, UK; 18 Imperial College London, London, UK; 19 Royal Cornwall Hospital Trust, Truro, UK; 20 Kent Oncology Centre, Maidstone and Tunbridge Wells NHS Trust, Maidstone, UK; 21 Blood Cancer UK, London, UK; 22 University College London, London, UK; 23 University Hospital Southampton NHS Foundation Trust, Southampton, UK; 24 National Cancer Research Institute Consumer Forum, London, UK; 25 Wye Valley NHS Trust, Hereford, UK; 26 National Health Service, London, UK; 27 University Hospitals Coventry and Warwickshire NHS Trust, Coventry, UK; 28 Royal College of Physicians, London, UK; 29 Guy’s and St Thomas’ NHS Foundation Trust, London, UK; 30 University of Bristol, Bristol, UK; 31 University College London Hospitals NHS Foundation Trust, London, UK; 32 Norfolk and Norwich University Hospitals NHS Foundation Trust, Norwich, UK; 33 Nuffield Department of Surgical Sciences, University of Oxford, Oxford, UK; 34 Sandwell and West Birmingham Hospitals NHS Trust, Birmingham, UK; 35 Royal United Hospital Bath NHS Trust, Bath, UK; 36 Institute of Immunology and Immunotherapy, University of Birmingham, Birmingham, UK; 37 Churchill Hospital, Oxford University Hospitals NHS Foundation Trust, Oxford, UK; 38 William Harvey Research Institute, London, UK; 39 University of Southampton, Southampton, UK

**Keywords:** Cancer, Outcomes research

## Abstract

Patients with cancer are at increased risk of hospitalisation and mortality following severe acute respiratory syndrome coronavirus 2 (SARS-CoV-2) infection. However, the SARS-CoV-2 phenotype evolution in patients with cancer since 2020 has not previously been described. We therefore evaluated SARS-CoV-2 on a UK populationscale from 01/11/2020-31/08/2022, assessing case-outcome rates of hospital assessment(s), intensive care admission and mortality. We observed that the SARS-CoV-2 disease phenotype has become less severe in patients with cancer and the non-cancer population. Case-hospitalisation rates for patients with cancer dropped from 30.58% in early 2021 to 7.45% in 2022 while case-mortality rates decreased from 20.53% to 3.25%. However, the risk of hospitalisation and mortality remains 2.10x and 2.54x higher in patients with cancer, respectively. Overall, the SARS-CoV-2 disease phenotype is less severe in 2022 compared to 2020 but patients with cancer remain at higher risk than the non-cancer population. Patients with cancer must therefore be empowered to live more normal lives, to see loved ones and families, while also being safeguarded with expanded measures to reduce the risk of transmission.

## Introduction

The SARS-CoV-2 pandemic has had a significant effect on cancer care globally since 2020. People with cancer are at heightened risk compared to the non-cancer population due to their increased propensity to infections, and there is evidence of poor immunological responses to COVID-19 vaccines and boosters^1–9^. Additionally, population scale studies have identified that patients with cancer experience waning immunity following vaccination to a much greater extent than the general population^10^.

Cancer centres in the United Kingdom have been at the vanguard of the global response to COVID-19, rapidly implementing new measures such as COVID-19 screening tests for patients undergoing cancer treatment^11^, implementing shielding (also known as isolation measures) when rates were high in the pre-vaccination era^12^, driving access to intensive care units^13^, promoting vaccination and vaccination boosters^10^, delivering access to diagnostics like antibody testing^14^, and implementing early treatment programmes with antivirals^15^.

It is widely acknowledged that the risk from SARS-CoV-2 is heterogenous. For people living with cancer, the risks are dependent on a combination of intrinsic patient factors, cancer factors (subtypes and treatments), COVID-19 measures (primary vaccination, boosters, early treatment programmes, access to intensive care units), and additionally by external factors such as the virulence of SARS-CoV-2 variants. The interactions of these factors vary over time as pandemics evolve, with changes to both the intrinsic and extrinsic risk factors. To date, there have been no population-scale analyses reporting on how the COVID-19 phenotype has evolved since 2020 in patients with cancer. Contemporary, accurate evaluations of levels of protection in patients with cancer are required as healthcare systems develop strategies for living with SARS-CoV-2 as an endemic disease. This information is also crucial for individual discussions between patients and their oncologists to adequately inform cancer treatment options.

There have been a small number of reports of severe COVID-19 outcomes in patients with cancer during 2022 when Omicron became the predominant circulating SARS-CoV-2 variant. In a study from Europe of 365 patients with cancer, the majority of whom were vaccinated against COVID-19, the case- hospitalisation rate was 24.4% with a 28-day case mortality of 13.1%^16^. In a subsequent study from the United States of 285 patients with cancer, of which 72% were vaccinated, a case mortality rate of 4.9% was observed^17^. Although on a small-scale, this indicates changes in clinical severity linked to circulating SARS-CoV-2 variants when compared to clinical outcomes of individuals earlier in the COVID-19 pandemic^18,19^.

The UK Coronavirus Cancer Programme (UKCCP) has been responsible for providing outcome analyses and severity metrics in the United Kingdom since March 2020. In this analysis, we report on case-outcome rates, including hospital assessment, hospitalisation, intensive care admission and case-mortality rates in patients with cancer, and how these have changed over time. This evaluation provides the largest global granular analyses of the complex interaction between intrinsic patient factors and severe COVID-19 outcomes from the start of the pandemic using our UK population-scale COVID-19 cancer dataset.

## Results

### Study dataset

During the study evaluation from 1st November 2020 to 31st August 2022, 198,819 positive SARS-CoV-2 polymerase chain reaction (PCR) and lateral flow device (LFD) tests were identified from individuals identified in the national cancer registry, corresponding to 127,322 individual infections. In the non-cancer population, 18,188,573 positive tests from 15,801,004 individual infections were identified. Within the cancer cohort, 39,033 SARS-CoV-2 tests were associated with a hospital assessment, 28,061 with inpatient hospitalisation, 2,168 with intensive care admission and 15,278 with SARS-CoV-2 mortality (Supplementary Table 2). Patient demographics including age, sex, ethnicity, deprivation and vaccination status were captured for each cohort and are outlined in Supplementary Table 3.

### Temporal changes in SARS-CoV-2 phenotype

**Figure 1:**
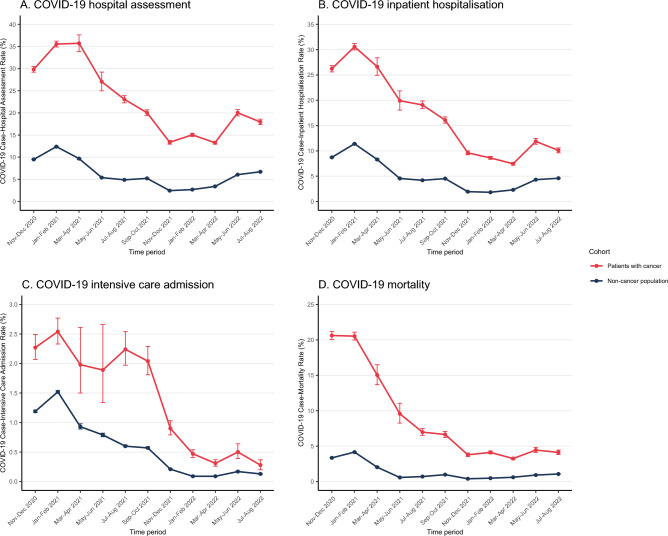
Temporal trends of case-outcome rates between patients with cancer and the non-cancer population in England between November 2020 and August 2022. Case-outcome rates (%) with 95% confidence intervals are shown at 2-month intervals for COVID-19 hospital assessment (**A**), inpatient hospitalisation (**B**), intensive care admission (**C**) and mortality (D).

We observed that during the study period from November 2020 to August 2022, the disease phenotype of the SARS-CoV-2 virus became less severe in both patients with cancer and the non-cancer population. COVID-19 hospital assessment and hospitalisation rates in patients with cancer decreased from 35.73% (884/2474) and 30.58% (6185/20,228) in early 2021 to 13.26% (5606/42,270) and 7.45% (3149/42,270) by Spring 2022, respectively (Supplementary Table 4). Similar drops in COVID-19 hospital assessment and hospitalisation rates were also noted in the non-cancer population (Fig. 1a-b, Supplementary Table 4). For both patients with cancer and the non-cancer population, COVID-19 case-intensive care admission rates of < 0.5% (131/42,270 and 2926/3,238,009, respectively) were observed in Spring 2022 as opposed to 2.54% (514/20,228) and 1.52% (17,812/1,173,794) in early 2021, respectively (Fig. 1c, Supplementary Table 4). The COVID-19 case-mortality rate in patients with cancer decreased from 20.62% (3961/19,209) in late 2020/early 2021 to 3.25% (1374/42,270) by spring 2022 with a corresponding decrease from 4.16% (48,839/1,173,794) to 0.40% (14,175/3,543,980) in the non-cancer population (Fig. 1d, Supplementary Table 4). Over the course of the study period, uptake of one or more COVID-19 vaccine dose(s) increased to over 90% for both the cancer and non-cancer population (Supplementary Fig. 1, Supplementary Table 5).

### SARS-CoV-2 phenotype in cancer subgroups in 2022

**Figure 2:**
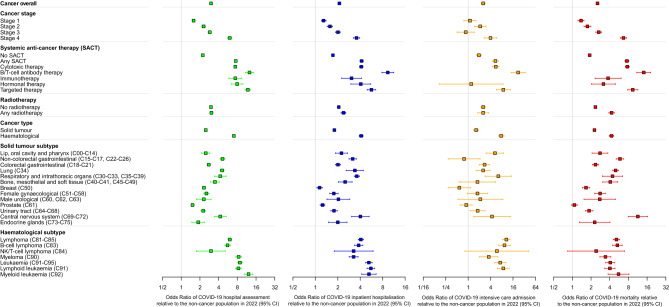
Relative risk of severe clinical outcomes in subgroups of patients with cancer compared to the non-cancer population following a COVID-19 infection between January 2022 and August 2022. Multivariable logistic regression models adjusting for age, sex, ethnicity and deprivation show the relative risk of COVID-19 hospital assessment (green), inpatient hospitalisation (blue), intensive care admission (orange) and mortality (red). Odds ratios are used to approximate relative risk with 95% confidence intervals. Corresponding ICD-10 codes for specific primary tumour subtypes are listed in brackets.

Having identified temporal trends in COVID-19 phenotype throughout the pandemic, we sought to understand the relative risk of these severe COVID-19 outcomes in the cancer cohort compared to the non-cancer population in 2022 by means of logistic regression models. We observed that the rate of hospital assessment was 5.34× (95% CI: 5.23–5.45) higher for patients with cancer with a 4.22× (95% CI: 4.11–4.33) higher rate of inpatient hospitalisation (Supplementary Fig. 2, all *p* < 0.00001). When adjusting for clinically relevant demographics including age, sex, ethnicity and deprivation, the relative risk of COVID-19 hospital assessment and inpatient hospitalisation in patients with cancer were 3.02× (95% CI 2.95–3.08) and 2.10× (95% CI 2.04–2.16), respectively (Fig. 2, all *p* < 0.00001). Similarly, the relative risk of COVID-19 intensive care admission and mortality within patients with cancer was 2.53× (95% CI 2.24–2.86) and 2.54× (95% CI 2.44–2.65) greater compared to the non-cancer population, respectively, when adjusted for age, sex, ethnicity and deprivation (Fig. 2, Supplementary Fig. 2, all *p* < 0.00001, Supplementary Table 6).

We then assessed the relative risks for subgroups of patients with cancer, compared to the non-cancer population. Our cancer subgroups included cancer stage, cancer treatment and cancer subtype as previously reported^12,13^. For severe COVID-19 outcomes (hospital assessment, inpatient hospitalisation, intensive care admission, COVID-19 mortality), the most notable subgroups associated with significantly higher risk were individuals with blood cancer (particularly leukaemia), receipt of SACT (cytotoxic therapy, B/T cell antibody, targeted therapy) and stage 4 cancer (Fig. 2, Supplementary Fig. 2).

### Personalised SARS-CoV-2 phenotype variations over time

Having identified differences in COVID-19 outcomes between different subgroups in 2022, we performed a more granular assessment of case-outcome rates across the COVID-19 pandemic. Building on previous analyses of COVID-19 outcomes, we calculated case-outcome rates for individual cancer subtypes across different age groups and sex in late 2020, 2021 and 2022^13^.

**Figure 3:**
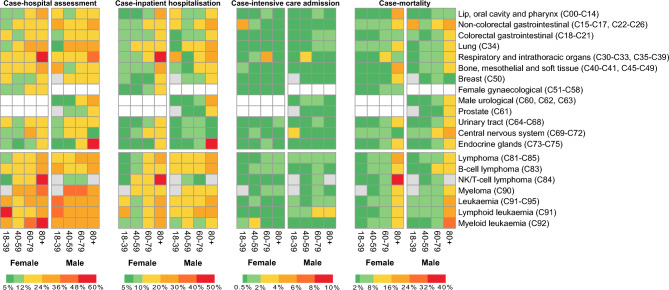
Case-outcome rates of solid and haematological cancer subtypes by age group and sex (female or male) following a COVID-19 infection in 2022. Case-outcome rates for COVID-19 hospital assessment, inpatient hospitalisation, intensive care admission and mortality are shown for each cancer subtype and are colour coded according to the scales below each heatmap. Grey boxes denote < 10 SARS-CoV-2 tests within a subgroup. White boxes denote < 10 SARS-CoV-2 tests within sex-specific cancer subtypes (female gynaecological, male urological, prostate cancers). Corresponding ICD-10 codes for specific primary tumour subtypes are listed in brackets.

We observed that case-outcome rates across the majority of cancer subtypes were lower in 2022, corresponding with the Omicron wave, when the majority of patients with cancer had received one or more COVID-19 vaccine dose, compared to both 2020 and 2021 (Supplementary Fig. 3). Individuals with haematological malignancies were at greater risk than those with solid organ cancers, with elevated risks also observed (though to a lesser extent) in those with lung cancers (Fig. 3, Supplementary Fig. 3).

Across the cancer cohort in 2022, patient age corresponded with greater differences in case-mortality rates than primary tumour subtype (Fig. 3). In 2022, the case-mortality rate for patients with cancer aged 80+ was 10.32% (1771/17,169, 95% CI 9.87–10.78) compared to 2.83% (2781/98,117, 95% CI 2.73–2.94) for those aged 18–79. The case-mortality rate for individuals with any haematological malignancy was 5.68% (945/16,649, 95% CI 5.33–6.04).

## Discussion

This UKCCP analysis has comprehensively described the evolution of the SARS-CoV-2 disease phenotype in patients with cancer within the UK. This population-scale study enables patients and clinicians to better understand risks from SARS-CoV-2. The disease phenotype is now different from what was experienced by patients with cancer and treating physicians in 2020 and likely to reflect global trends. Our analyses have identified that severe SARS-CoV-2 outcomes (hospital assessment, inpatient hospitalisation, intensive care and mortality) are now less frequent. Fewer patients are hospitalised, and many may be managed in the community. Nevertheless, patients with cancer remain at elevated risk of these outcomes compared to the non-cancer population.

The reduction in the severity of disease phenotype is likely attributable to the great strides taken by health care professionals to adapt cancer care, including access to vaccination and reconfigurations of treatment programmes and wider healthcare services. This includes the rapid early-stage roll-out of SARS-CoV-2 vaccines by UK public health bodies and the National Health Service to clinically vulnerable individuals including people living with cancer. More recently, antiviral and monoclonal therapies have been developed and utilised, further improving clinical outcomes^20–23^. These measures have likely made cancer centres better protected from respiratory pathogens than at any time in history.

However, the risks of severe COVID-19 outcomes are still significantly higher in patients with cancer than the general population and it is therefore important to maintain access to protective measures for this group. The excess risks associated with the receipt of SACT in our population is at clear odds to papers that were published in 2020 in the pre-vaccination era^24,25^. B and T cell-depleting therapies were associated with increased risk. This is possibly the result of more comprehensive evaluations of COVID-19 outcomes within patients receiving SACT and corresponds with evidence of lower vaccine effectiveness and post-vaccination SARS-CoV-2 antibody responses in patients receiving SACT^10,26,27^. The elevated risks of severe COVID-19 outcomes therefore need to be weighed against the oncological benefits of cancer treatment. We hope that our dataset will enable patients and clinicians to understand the relative risks and benefits of cancer treatment in an evolving pandemic landscape and allow for informed joint decision-making.

The trends of SARS-CoV-2 outcome severity must be monitored and further efforts to reduce excess risk experienced by patients with cancer remain important. Many countries are now employing new approaches such as pre-exposure prophylactic antibody therapies and immunisations to provide a critical boost for those who derive lower levels of benefit from vaccination. Infection prevention in patients with cancer is important; concerns around treating COVID-19-positive patients will inevitably lead to delays in delivering effective cancer care and ultimately poorer cancer outcomes. However, the effectiveness of prophylactic antibody therapies needs to be assessed with different disease variants of SARS-CoV-2, and their ongoing benefit evaluated.

There are a few limitations to this study. The first is that we have reported on case severity and not absolute number of events. Unrestrained community transmission of SARS-CoV-2 with a low severe event rate will lead to more cases of severe events in the population, whilst the risk to given individuals remains low. In addition, clinical guidelines for hospital and intensive care admissions for patients with cancer were altered during the pandemic for managing healthcare capacity and infection control which might have impacted admission of subgroups of cancer patients, possibly including those with advanced-stage disease^28,29^. Secondly, we acknowledge that further iterations and evolution of the multivariable models could be performed to incorporate additional clinically-important covariates, such as vaccination status and time from most recent vaccine, which could be informative for clinical decision-making. Furthermore, our cancer registry takes time to accrue cases and the most recent diagnoses are not recorded. This will therefore be an underestimate of COVID-19 risk, though this will occur in both populations of interest. Finally, we know there is clinical utility of diagnostic tests such as antibody responses in terms of forecasting risk in cancer cohorts and this was not assessed in this analysis^30^.

To summarise, in patients with cancer, the risk of severe COVID-19 events is at its lowest since 2020. Following interventions by the oncology community, the disease phenotype is now markedly less severe for most patients with cancer compared to previous years. Further work can and should be done to reduce the excess risk to patients with cancer and provide similar levels of COVID-19 protection as the wider population. This work can be expanded to other at-risk populations, for example immunosuppressed post-transplant patients. A renewed focus on strategies to maximise quality of living as well as a focus back to effective cancer care and research are more important than ever. In combination, these measures will allow healthcare systems to deliver successful cancer research programmes and safeguard the future for those who have developed or are at risk of developing cancer.

## Online methods

### Study setting

The UKCCP is one of the United Kingdom’s longest running pandemic responses with a mission to safeguard, evaluate and protect patients with cancer, (www.ukcovidcancerprogramme.org). This project was a population-based study of COVID-19 outcomes in patients with cancer from the study period of 1st November 2020 to 31st August 2022, initiated to define the disease phenotype in the highest clinical risk groups. The study period includes significant milestones over the course of the pandemic, including the start of the COVID-19 vaccination programme (December 2020), the COVID-19 booster vaccines (September 2021), SARS-CoV-2 Delta variant wave (April 2021–December 2021), the start of the SARS-CoV-2 Omicron variant wave (December 2021–April 2022) and the availability of community antiviral treatment following recent infection (June 2022).

### Study design and population

The study was performed as a population-scale case–control evaluation of clinical outcomes following a SARS-CoV-2 infection in patients with cancer and the non-cancer population. The study population contains all positive SARS-CoV-2 polymerase chain reaction (PCR) and lateral flow device (LFD) test results from England during the study period. The cancer cohort comprises adults (18 years or older) who underwent SARS-CoV-2 testing, identified from Public Health England’s rapid registration national cancer dataset between 1st January 2018 and 30th April 2021. A control population cohort was constructed from SARS-CoV-2 tests from adults who were not contained within this national cancer dataset. The study was designed as a public health surveillance analysis to support rapid clinical decision making during the pandemic in accordance with the UK Policy Framework for Health and Social Care Research. The project was supported by the UK Health Security Agency (UKHSA) with ethical approval from the Health Research Authority (20/WA/0181). The corresponding authors and senior author had final responsibility for the decision to submit for publication.

### Data and sampling

NHS England used PCR testing for those with COVID-19 symptoms, and LFD testing, also known as antigen-detecting rapid diagnostic testing, for identification of asymptomatic cases. Furthermore, in the NHS, infection and prevention control measures in secondary care required COVID-19 testing of asymptomatic inpatients and outpatients prior to many procedures or treatments. From 1st April 2022, guidelines for COVID-19 testing in the community were updated to end free, universal symptomatic and asymptomatic testing for the general public in England but continue testing provision for eligible clinically-vulnerable individuals^31^. Identification of patient-level COVID-19 test results, including from community and hospital testing, were obtained from the second-generation surveillance system (SGSS). Corresponding hospital and intensive-care admission records were obtained from the Secondary Use Statistics (SUS) datasets and COVID-19 vaccination records from the National Immunisation Management Service (NIMS) for both the cancer cohort and non-cancer population control. Data linkage required exact matching of NHS ID at the patient level between each dataset, and each data point corresponds to a single SARS-CoV-2 test. All data was anonymised prior to data analysis and no patient-identifiable features are included within the manuscript in accordance with relevant guidelines and regulations to maintain data security and patient confidentiality.

The UKCCP dataset comprises information including age, sex, ethnicity, vaccination status and deprivation, with geographical location being used to determine the index of multiple deprivation (IMD), a national indicator of deprivation^32^. For patients with cancer, the dataset contains information about cancer stage and subtype, receipt of radiotherapy and systemic anti-cancer treatments (SACT). SACT is an umbrella term of cancer treatments including cytotoxic agents (chemotherapy), targeted, immune or hormonal therapies. SACT was also divided into five different classes (cytotoxic, B or T cell antibody, targeted, immunotherapy, hormonal) with treatment classification outlined in Supplementary Table 1. Variables were either binary or grouped with age categorised into age bands (18–39, 40–59, 60–79, 80+) to align with previous UKCCP studies^13,24^.

### Statistical Analysis

The co-primary outcomes of the study were COVID-19 case-outcome rates including COVID-19 hospital assessment, COVID-19 inpatient hospitalisation, COVID-19 intensive care admission and COVID-19 mortality. A COVID-19 hospital assessment was defined as a secondary care encounter from − 1 to + 14 days of a positive SARS-CoV-2 test. A COVID-19 inpatient hospitalisation was defined as a hospitalisation episode lasting more than one day. A COVID-19 intensive care admission was an intensive care admission following a COVID-19 hospitalisation. COVID-19 mortality was defined as any death up to 28 days of a positive SARS-CoV-2 test, in keeping with standard COVID-19 deaths reported by UK Office for National Statistics^10^.

Univariable and multivariable logistic regression were utilised to assess the relative risk of COVID-19 outcomes in subgroups of patients with cancer compared to the non-cancer population. This analysis was performed in 2022 when Omicron had become the predominant SARS-CoV-2 variant circulating in the population. Adjustments were made for clinically important covariates of patient age, sex, ethnicity and deprivation which may act as confounders and/or effect modifiers for analysing clinical outcomes following a SARS-CoV-2 infection and performed as per our previous analyses^10,26^. Univariable analyses were also performed to determine absolute COVID-19 case-outcome rates, with cancer subtype subdivided by patient age and sex and performed as per our previous analyses^13^.

Within the cancer cohort, cancer subgroup analyses were assessed with ICD-10 subtype codes^33^, cancer stage, receipt of systemic anti-cancer therapy (SACT) and/or radiotherapy. Cancer treatments (SACT or radiotherapy) were evaluated as to whether the individual had received these treatments.

## Data availability and patient consent

Individual participant data was utilised in the study, for which informed patient consent was obtained. All data was anonymised prior to data analysis and no patient-identifiable features are included within the manuscript in accordance with relevant guidelines and regulations. In order to comply with data privacy laws, data from this study, including individual participant data is not available for sharing. Data field definition within the data dictionary are available by reasonable request. The privacy statement for individuals performing coronavirus testing provided by the UK Health Security Agency (UKHSA) and Department for Health and Social care (DHSC) is available here: https://www.gov.uk/government/publications/phe-privacy-information/privacy-information.

## Supplementary Information

Supplementary Information 1.

Supplementary Information 2.

Supplementary Information 3.

Supplementary Information 4.

Supplementary Information 5.
