# Supplementary material for: A population-scale temporal case–control evaluation of COVID-19 disease phenotype and related outcome rates in patients with cancer in England (UKCCP)
Source: Sci Rep. 2023 Jul 25;13:11327. doi: 10.1038/s41598-023-36990-9 (PMC10368624; doi:10.1038/s41598-023-36990-9)
Supplement: Supplementary file 2 — Supplementary Information 2. [file 41598_2023_36990_MOESM2_ESM.docx]

**Supplementary Tables**

SUPPLEMENTARY TABLE 1 – SEPARATE EXCEL FILE

***Supplementary Table 1:*** *Classification of systemic anti-cancer therapies into five categories: cytotoxic agents, B/T cell antibody therapy, targeted therapy, immunotherapy and hormonal therapy.*

|  | Patients with cancer | | Non-cancer population | |
| --- | --- | --- | --- | --- |
| **Clinical Outcomes** | **Tests** | **Patients** | **Tests** | **Patients** |
| COVID-19 infection (overall cohort) | 198819 | 127322 | 18188573 | 15801004 |
| Hospital assessment | 39033 | 23264 | 837081 | 536566 |
| Inpatient hospitalisation | 28061 | 16261 | 671144 | 412087 |
| Intensive care admission | 2168 | 1136 | 68088 | 36162 |
| COVID-19 mortality | 15278 | 9580 | 184498 | 113043 |

***Supplementary Table 2:*** *Clinical outcomes of the UKCCP cancer cohort and non-cancer population control at the SARS-CoV-2 positive test level and individual patient level.*

| **Demographics** | **Patients with cancer** | **Non-cancer population** |
| --- | --- | --- |
|  |  |  |
| **Age group** |  |  |
| 18-19 | 389 (0.20%) | 591296 (3.25%) |
| 20-29 | 3143 (1.58%) | 3223020 (17.72%) |
| 30-39 | 8703 (4.38%) | 3781383 (20.79%) |
| 40-49 | 17635 (8.87%) | 3598039 (19.78%) |
| 50-59 | 36705 (18.46%) | 3200888 (17.60%) |
| 60-69 | 48417 (24.35%) | 1928813 (10.60%) |
| 70-79 | 51591 (25.95%) | 1088001 (5.98%) |
| 80-89 | 26373 (13.26%) | 559060 (3.07%) |
| 90-99 | 5788 (2.91%) | 208561 (1.15%) |
| 100+ | 75 (0.04%) | 9512 (0.05%) |
|  |  |  |
| **Sex** |  |  |
| Female | 98452 (49.52%) | 10356254 (56.94%) |
| Male | 100367 (50.48%) | 7830844 (43.05%) |
|  |  |  |
| **Ethnicity** |  |  |
| Asian/Asian British | 8053 (4.05%) | 1396446 (7.68%) |
| Black/Black British | 4707 (2.37%) | 504064 (2.77%) |
| White/White British | 181765 (91.42%) | 15253698 (83.86%) |
| Mixed or Other Ethnicity | 1725 (0.87%) | 311899 (1.71%) |
|  |  |  |
| **Indices of Deprivation (IMD)** |  |  |
| Low (IMD 1-3) | 45788 (23.03%) | 5007809 (27.53%) |
| Medium (IMD 4-7) | 75897 (38.17%) | 7248607 (39.85%) |
| High (IMD 8-10) | 62861 (31.62%) | 5514627 (30.32%) |
|  |  |  |
| **SARS-CoV-2 Vaccination** |  |  |
| Vaccinated (1+ Dose) | 156187 (78.56%) | 14028232 (77.13%) |
| Unvaccinated (0 Doses) | 42632 (21.44%) | 4160341 (22.87%) |
|  |  |  |

***Supplementary Table 3:*** *Baseline demographics of the UKCCP cancer cohort and non-cancer population control at the SARS-CoV-2 positive test level.*

| Hospital Assessment | | | | |
| --- | --- | --- | --- | --- |
|  | *Patients with cancer* | | *Non-cancer population* | |
| **Time Period** | **Outcome/Total (n/N)** | **Outcome % (95% CI)** | **Outcome/Total (n/N)** | **Outcome % (95% CI)** |
| Nov-Dec 2020 | 5732/19209 | 29.84% (29.20-30.49) | 103983/1094943 | 9.50% (9.44-9.55) |
| Jan-Feb 2021 | 7189/20228 | 35.54% (34.88-36.20) | 145402/1173794 | 12.39% (12.33-12.45) |
| Mar-Apr 2021 | 884/2474 | 35.73% (33.87-37.64) | 16146/166778 | 9.68% (9.54-9.82) |
| May-Jun 2021 | 458/1693 | 27.05% (24.99-29.22) | 16460/304909 | 5.40% (5.32-5.48) |
| Jul-Aug 2021 | 2420/10485 | 23.08% (22.28-23.90) | 72421/1481046 | 4.89% (4.86-4.92) |
| Sep-Oct 2021 | 2733/13638 | 20.04% (19.38-20.72) | 68613/1314307 | 5.22% (5.18-5.26) |
| Nov-Dec 2021 | 3154/23592 | 13.37% (12.94-13.81) | 86812/3543980 | 2.45% (2.43-2.47) |
| Jan-Feb 2022 | 5731/38076 | 15.05% (14.70-15.41) | 114847/4273918 | 2.69% (2.67-2.70) |
| Mar-Apr 2022 | 5606/42270 | 13.26% (12.94-13.59) | 109938/3238009 | 3.40% (3.38-3.42) |
| May-Jun 2022 | 2401/11988 | 20.03% (19.32-20.75) | 43897/723862 | 6.06% (6.01-6.12) |
| Jul-Aug 2022 | 2725/15166 | 17.97% (17.36-18.59) | 58562/873027 | 6.71% (6.66-6.76) |
|  |  |  |  |  |
| Inpatient Hospitalisation | | | | |
|  | *Patients with cancer* | | *Non-cancer population* | |
| **Time Period** | **Outcome/Total (n/N)** | **Outcome % (95% CI)** | **Outcome/Total (n/N)** | **Outcome % (95% CI)** |
| Nov-Dec 2020 | 5036/19209 | 26.22% (25.60-26.84) | 95525/1094943 | 8.72% (8.67-8.78) |
| Jan-Feb 2021 | 6185/20228 | 30.58% (29.95-31.21) | 133795/1173794 | 11.40% (11.34-11.46) |
| Mar-Apr 2021 | 659/2474 | 26.64% (24.93-28.41) | 13845/166778 | 8.30% (8.17-8.43) |
| May-Jun 2021 | 337/1693 | 19.91% (18.07-21.87) | 13878/304909 | 4.55% (4.48-4.63) |
| Jul-Aug 2021 | 2001/10485 | 19.08% (18.34-19.85) | 61994/1481046 | 4.19% (4.15-4.22) |
| Sep-Oct 2021 | 2193/13638 | 16.08% (15.47-16.71) | 59450/1314307 | 4.52% (4.49-4.56) |
| Nov-Dec 2021 | 2267/23592 | 9.61% (9.24-9.99) | 68884/3543980 | 1.94% (1.93-1.96) |
| Jan-Feb 2022 | 3280/38076 | 8.61% (8.34-8.90) | 77858/4273918 | 1.82% (1.81-1.83) |
| Mar-Apr 2022 | 3149/42270 | 7.45% (7.20-7.70) | 74623/3238009 | 2.30% (2.29-2.32) |
| May-Jun 2022 | 1424/11988 | 11.88% (11.31-12.47) | 31203/723862 | 4.31% (4.26-4.36) |
| Jul-Aug 2022 | 1530/15166 | 10.09% (9.62-10.58) | 40089/873027 | 4.59% (4.55-4.64) |
|  |  |  |  |  |
| Intensive Care Admission | | | | |
|  | *Patients with cancer* | | *Non-cancer population* | |
| **Time Period** | **Outcome/Total (n/N)** | **Outcome % (95% CI)** | **Outcome/Total (n/N)** | **Outcome % (95% CI)** |
| Nov-Dec 2020 | 436/19209 | 2.27% (2.07-2.49) | 13028/1094943 | 1.19% (1.17-1.21) |
| Jan-Feb 2021 | 514/20228 | 2.54% (2.33-2.77) | 17812/1173794 | 1.52% (1.50-1.54) |
| Mar-Apr 2021 | 49/2474 | 1.98% (1.50-2.61) | 1553/166778 | 0.93% (0.89-0.98) |
| May-Jun 2021 | 32/1693 | 1.89% (1.34-2.66) | 2396/304909 | 0.79% (0.76-0.82) |
| Jul-Aug 2021 | 235/10485 | 2.24% (1.97-2.54) | 8943/1481046 | 0.60% (0.59-0.62) |
| Sep-Oct 2021 | 278/13638 | 2.04% (1.81-2.29) | 7521/1314307 | 0.57% (0.56-0.59) |
| Nov-Dec 2021 | 212/23592 | 0.90% (0.79-1.03) | 7563/3543980 | 0.21% (0.21-0.22) |
| Jan-Feb 2022 | 179/38076 | 0.47% (0.41-0.54) | 3966/4273918 | 0.09% (0.09-0.10) |
| Mar-Apr 2022 | 131/42270 | 0.31% (0.26-0.37) | 2926/3238009 | 0.09% (0.09-0.09) |
| May-Jun 2022 | 60/11988 | 0.50% (0.39-0.64) | 1263/723862 | 0.17% (0.17-0.18) |
| Jul-Aug 2022 | 42/15166 | 0.28% (0.20-0.37) | 1117/873027 | 0.13% (0.12-0.14) |
|  |  |  |  |  |
| COVID-19 Mortality | | | | |
|  | *Patients with cancer* | | *Non-cancer population* | |
| **Time Period** | **Outcome/Total (n/N)** | **Outcome % (95% CI)** | **Outcome/Total (n/N)** | **Outcome % (95% CI)** |
| Nov-Dec 2020 | 3961/19209 | 20.62% (20.05-21.20) | 36681/1094943 | 3.35% (3.32-3.38) |
| Jan-Feb 2021 | 4152/20228 | 20.53% (19.98-21.09) | 48839/1173794 | 4.16% (4.12-4.20) |
| Mar-Apr 2021 | 372/2474 | 15.04% (13.68-16.50) | 3384/166778 | 2.03% (1.96-2.10) |
| May-Jun 2021 | 162/1693 | 9.57% (8.26-11.06) | 1776/304909 | 0.58% (0.56-0.61) |
| Jul-Aug 2021 | 732/10485 | 6.98% (6.51-7.49) | 10585/1481046 | 0.71% (0.70-0.73) |
| Sep-Oct 2021 | 907/13638 | 6.65% (6.24-7.08) | 12877/1314307 | 0.98% (0.96-1.00) |
| Nov-Dec 2021 | 891/23592 | 3.78% (3.54-4.03) | 14175/3543980 | 0.40% (0.39-0.41) |
| Jan-Feb 2022 | 1568/38076 | 4.12% (3.92-4.32) | 20470/4273918 | 0.48% (0.47-0.49) |
| Mar-Apr 2022 | 1374/42270 | 3.25% (3.09-3.42) | 19701/3238009 | 0.61% (0.60-0.62) |
| May-Jun 2022 | 534/11988 | 4.45% (4.10-4.84) | 6679/723862 | 0.92% (0.90-0.94) |
| Jul-Aug 2022 | 625/15166 | 4.12% (3.82-4.45) | 9331/873027 | 1.07% (1.05-1.09) |

***Supplementary Table 4:*** *Temporal analysis of COVID-19 case-outcome rates in the UKCCP cancer cohort and non-cancer population control. Case-outcome rates (outcome %) were calculated in 2-month intervals from 1^st^ November 2020-31^st^ August 2022. 95% confidence intervals for case-outcome rates are provided in brackets.*

|  | **Patients with cancer** | | **Non-cancer population** | |
| --- | --- | --- | --- | --- |
| **Time period** | **Vaccinated/Total (n/N)** | **Uptake % (95% CI)** | **Vaccinated/Total (n/N)** | **Uptake % (95% CI)** |
| Nov-Dec 2020 | 115/19209 | 0.60% (0.50-0.72) | 6590/1094943 | 0.60% (0.59-0.62) |
| Jan-Feb 2021 | 3603/20228 | 17.81% (17.29-18.35) | 130256/1173794 | 11.10% (11.04-11.15) |
| Mar-Apr 2021 | 1607/2474 | 64.96% (63.05-66.81) | 58914/166778 | 35.32% (35.10-35.55) |
| May-Jun 2021 | 1379/1693 | 81.45% (79.53-83.23) | 160930/304909 | 52.78% (52.60-52.96) |
| Jul-Aug 2021 | 9493/10485 | 90.54% (89.96-91.08) | 1073603/1481046 | 72.49% (72.42-72.56) |
| Sep-Oct 2021 | 12859/13638 | 94.29% (93.89-94.67) | 1100684/1314307 | 83.75% (83.68-83.81) |
| Nov-Dec 2021 | 22376/23592 | 94.85% (94.56-95.12) | 3084237/3543980 | 87.03% (86.99-87.06) |
| Jan-Feb 2022 | 36704/38076 | 96.40% (96.20-96.58) | 3851883/4273918 | 90.13% (90.10-90.15) |
| Mar-Apr 2022 | 41431/42270 | 98.02% (97.88-98.14) | 3054173/3238009 | 94.32% (94.30-94.35) |
| May-Jun 2022 | 11759/11988 | 98.09% (97.83-98.32) | 680972/723862 | 94.07% (94.02-94.13) |
| Jul-Aug 2022 | 14861/15166 | 97.99% (97.75-98.20) | 825990/873027 | 94.61% (94.56-94.66) |

***Supplementary Table 5:*** *Temporal comparison of SARS-CoV-2 vaccination uptake within patients within cancer and the non-cancer population. Vaccination uptake was calculated as the proportion of SARS-CoV-2 tests taken by individuals who had received at least one SARS-CoV-2 vaccination dose at each temporal timepoint.*

|  | Univariable | | Multivariable adjusted | |
| --- | --- | --- | --- | --- |
| **Clinical Outcome** | **OR (95% CI)** | **p value** | **OR (95% CI)** | **p value** |
| Hospital assessment | 5.34 (5.23-5.45) | <0.00001 | 3.02 (2.95-3.08) | <0.00001 |
| Inpatient hospitalisation | 4.22 (4.11-4.33) | <0.00001 | 2.10 (2.04-2.16) | <0.00001 |
| Intensive care admission | 4.22 (3.76-4.74) | <0.00001 | 2.53 (2.24-2.86) | <0.00001 |
| COVID-19 mortality | 7.12 (6.85-7.40) | <0.00001 | 2.54 (2.44-2.65) | <0.00001 |

***Supplementary Table 6:*** *Relative risk of patients with cancer to severe clinical outcomes compared to the non-cancer population following a COVID-19 infection. Multivariable logistic regression models were adjusted for age, sex, ethnicity and deprivation.*

**Supplementary Figure Legends**

***Supplementary Figure 1: Time plot illustrating the proportion of tests from vaccinated individuals within patients with cancer and the non-cancer population.*** *The proportion of tests from vaccinated individuals was calculated at each timepoint between November 2020 and August 2022. Individuals were considered to be vaccinated if they have received at least one SARS-CoV-2 vaccination dose.*

***Supplementary Figure 2: Relative risk of subgroups of patients with cancer to severe clinical outcomes compared to the non-cancer population following a COVID-19 infection between January 2022 and August 2022.*** *Univariable logistic regression models adjusting for age, sex, ethnicity and deprivation show the relative risk of COVID-19 hospital assessment (green), inpatient hospitalisation (blue), intensive care admission (orange) and mortality (red). Relative risk to the non-cancer population is shown as Odds Ratios (OR) with 95% confidence intervals. Corresponding ICD-10 codes for specific primary tumour subtypes are listed in brackets.*

***Supplementary Figure 3: Case-outcome rates of solid and haematological cancer subtypes by age group and sex (female or male) following a COVID-19 infection in 2020, 2021 and 2022.*** *Case-outcome rates for COVID-19 hospital assessment, inpatient hospitalisation, intensive care admission and mortality are shown for each cancer subtype and are colour coded according to the scales below each heatmap. Grey boxes denote <10 SARS-CoV-2 tests within a subgroup. White boxes denote <10 SARS-CoV-2 tests within sex-specific cancer subtypes (female gynaecological, male urological, prostate cancers). Corresponding ICD-10 codes for specific primary tumour subtypes are listed in brackets.*
