# Supplementary figures and images for: A population-scale temporal case–control evaluation of COVID-19 disease phenotype and related outcome rates in patients with cancer in England (UKCCP)
Source: Sci Rep. 2023 Jul 25;13:11327. doi: 10.1038/s41598-023-36990-9 (PMC10368624; doi:10.1038/s41598-023-36990-9)

# COVID-19 vaccination uptake

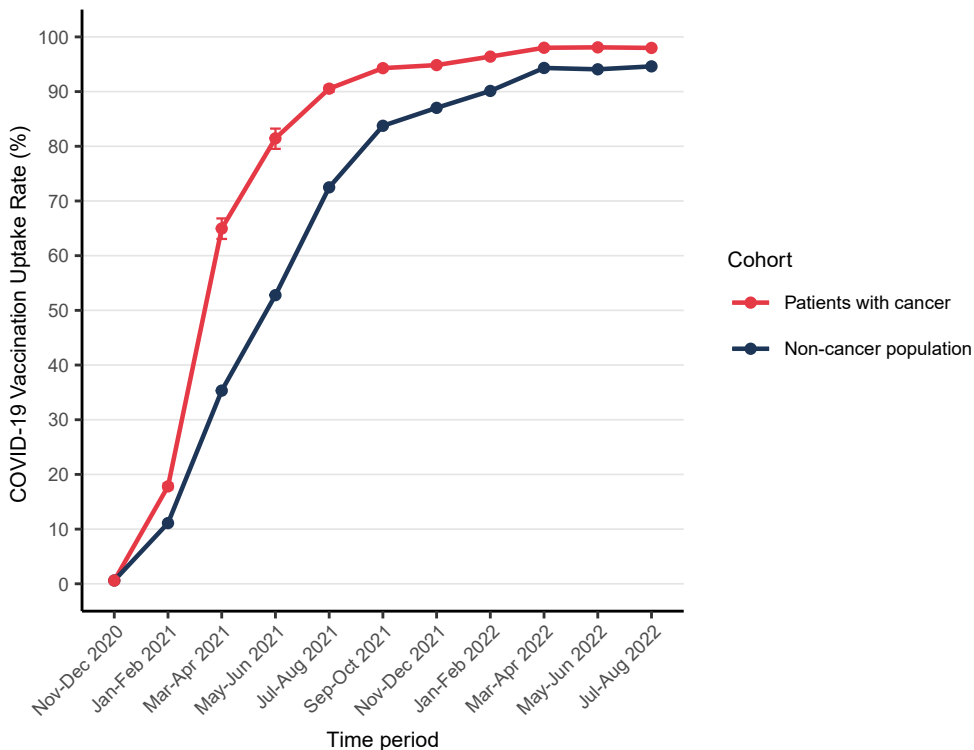

Supplement: Supplementary file 3 — Supplementary Information 3. [file 41598_2023_36990_MOESM3_ESM.pdf]

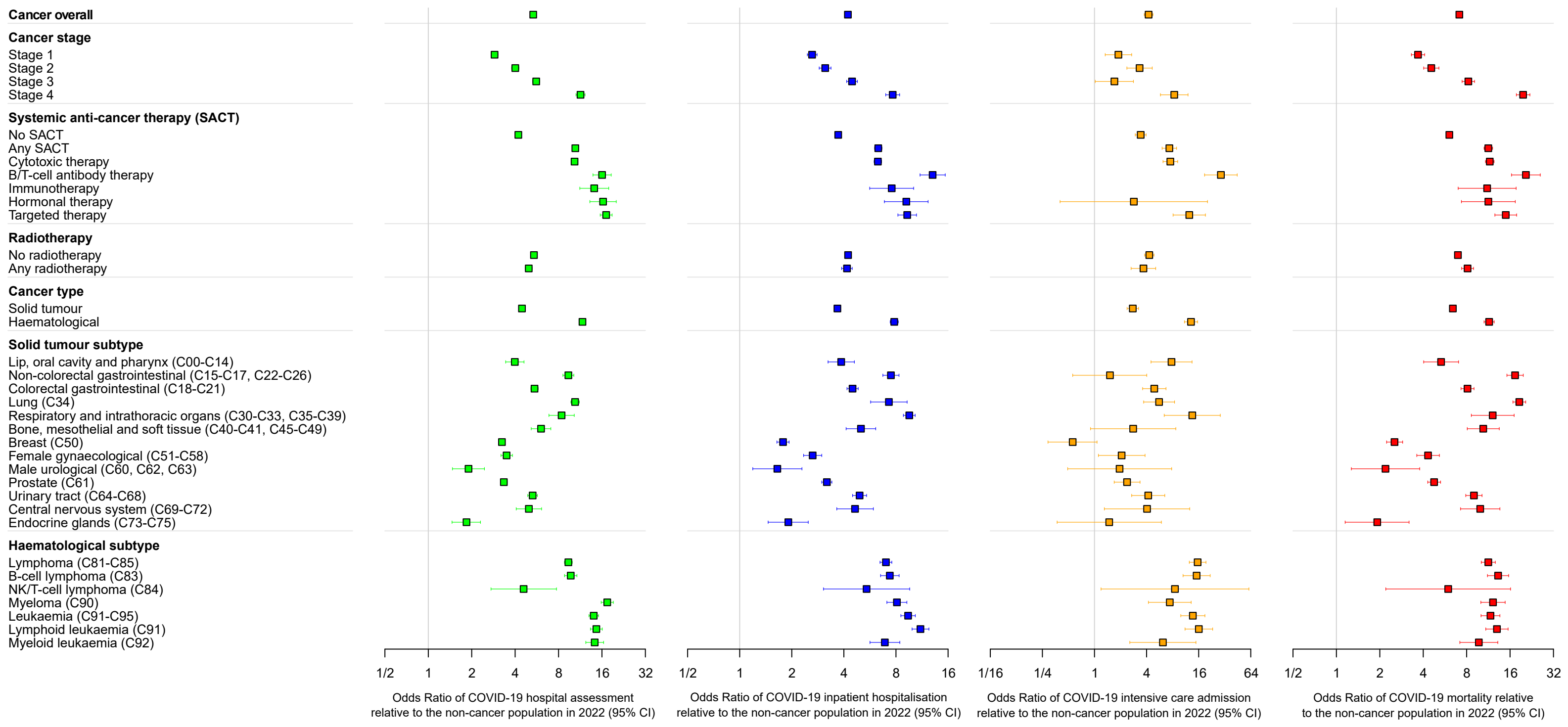

Supplement: Supplementary file 4 — Supplementary Information 4. [file 41598_2023_36990_MOESM4_ESM.pdf]
