# Supplementary material for: A population-scale temporal case–control evaluation of COVID-19 disease phenotype and related outcome rates in patients with cancer in England (UKCCP)
Source: Sci Rep. 2023 Jul 25;13:11327. doi: 10.1038/s41598-023-36990-9 (PMC10368624; doi:10.1038/s41598-023-36990-9)

Case-hospital assessment percentage by cancer subtype

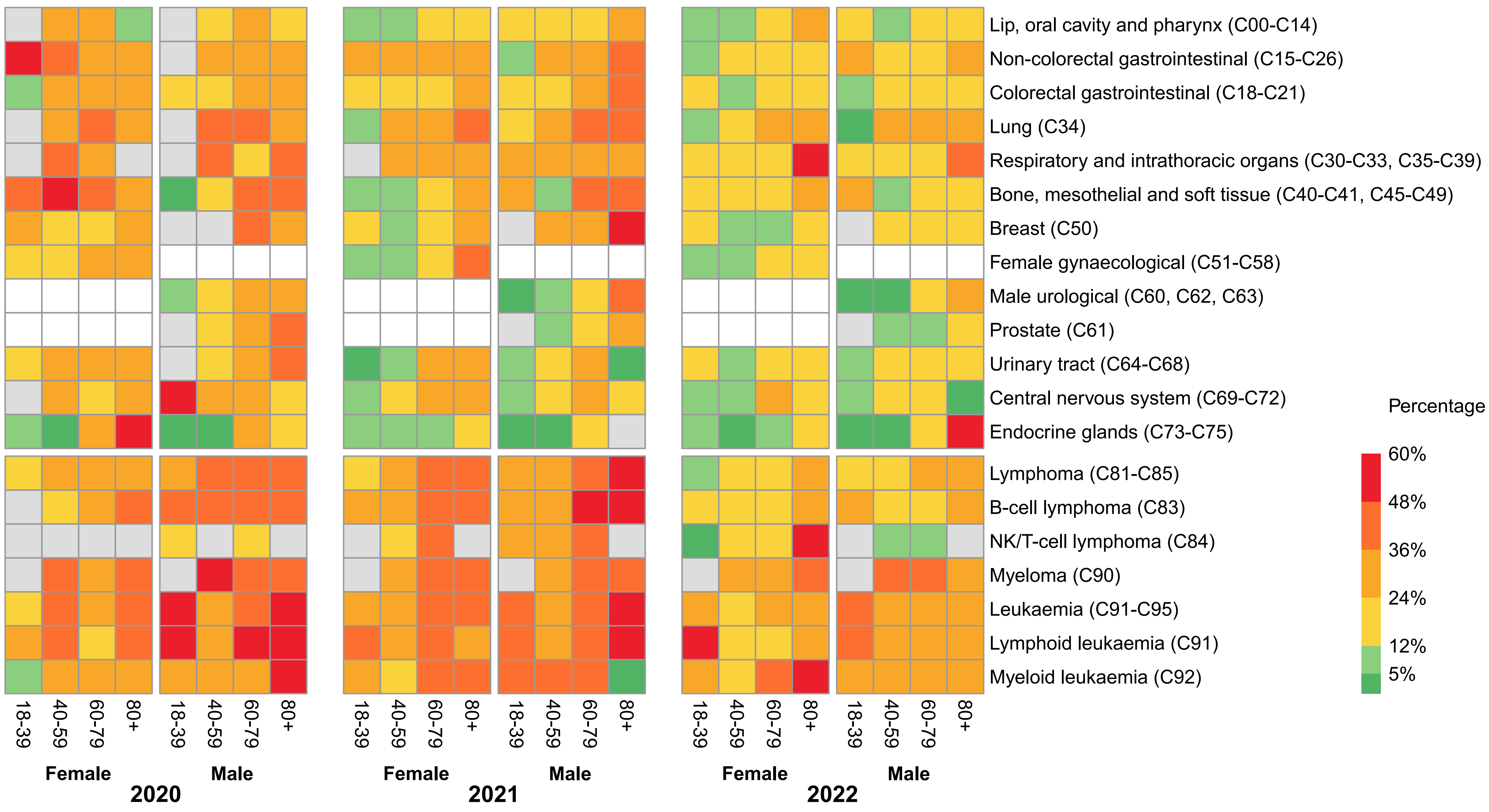

Case-inpatient hospitalisation percentage by cancer subtype

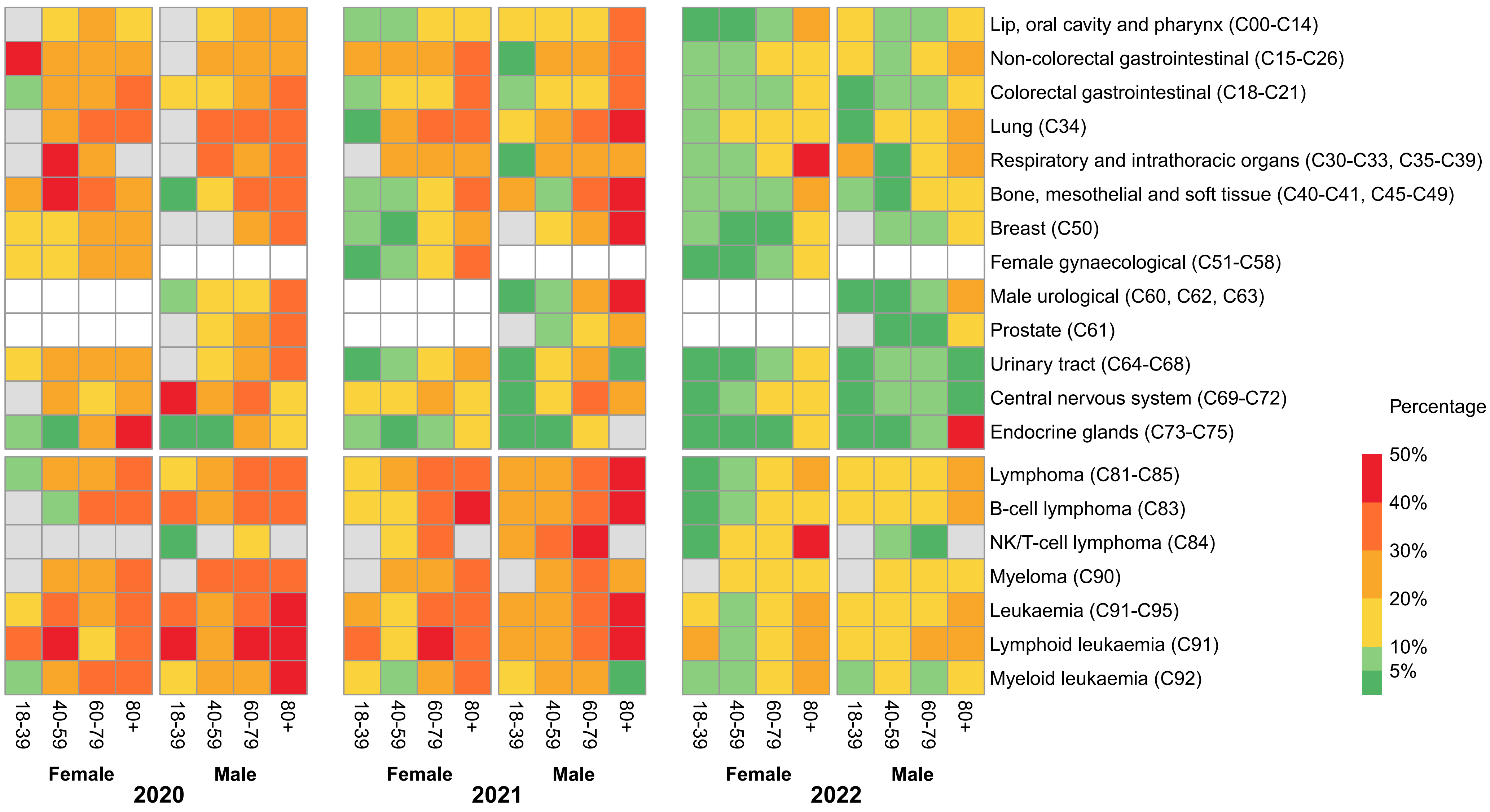

Case-intensive care admission percentage by cancer subtype

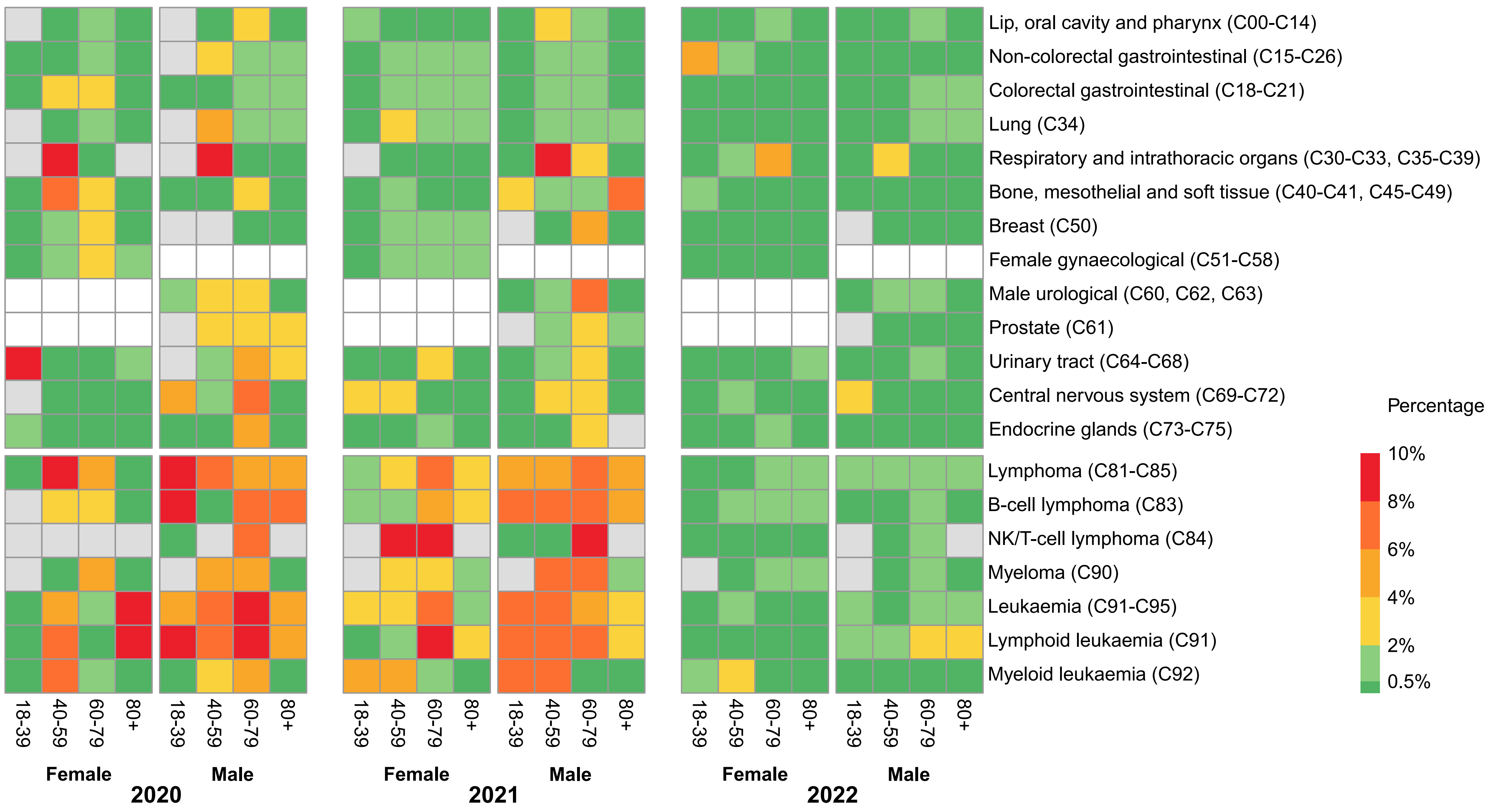

Case-mortality percentage by cancer subtype

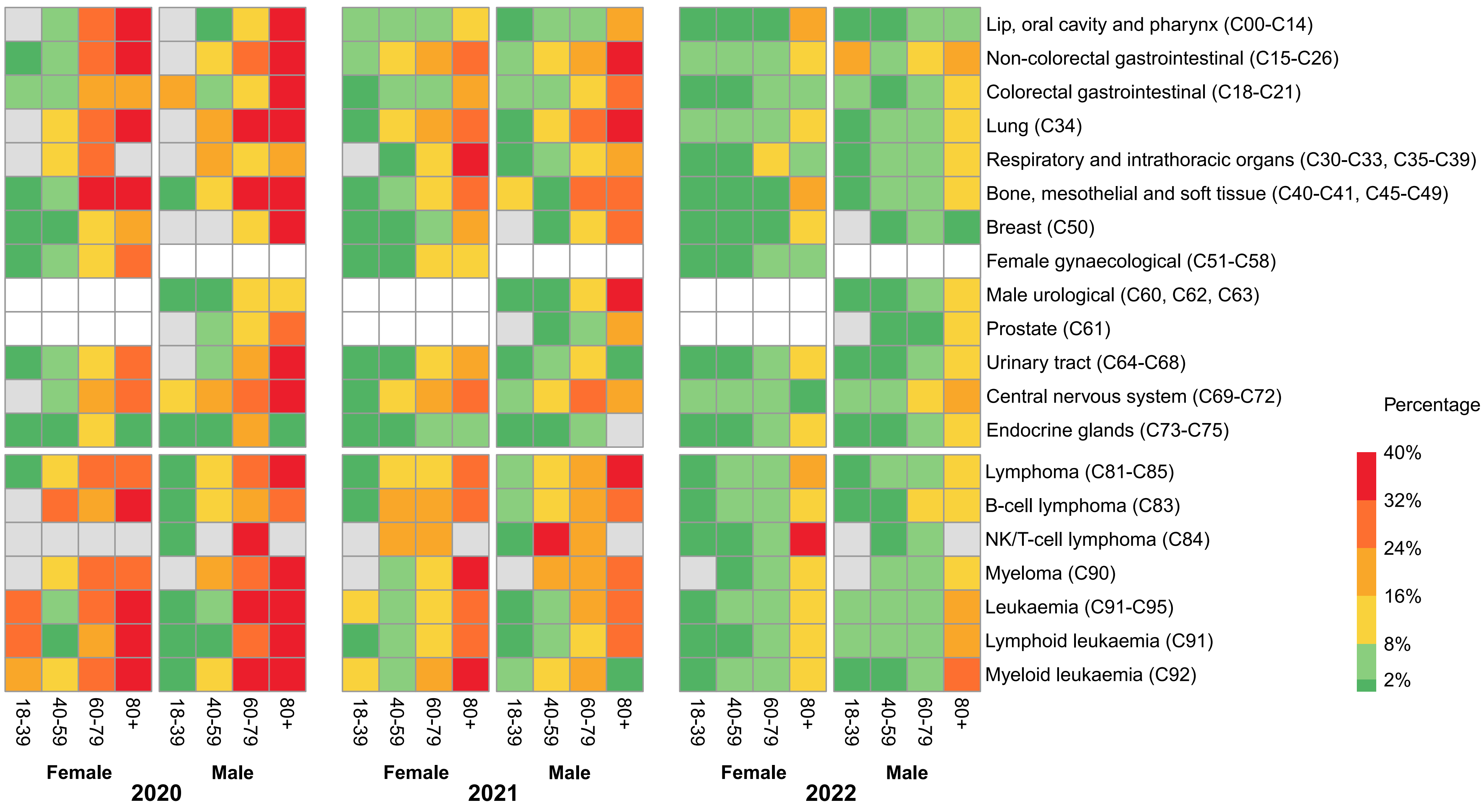

Supplement: Supplementary file 5 — Supplementary Information 5. [file 41598_2023_36990_MOESM5_ESM.pdf]
